# SNP Discovery through Next-Generation Sequencing and Its Applications

**DOI:** 10.1155/2012/831460

**Published:** 2012-11-22

**Authors:** Santosh Kumar, Travis W. Banks, Sylvie Cloutier

**Affiliations:** ^1^Department of Plant Science, University of Manitoba, Winnipeg, MB, Canada R3T 2N2; ^2^Department of Applied Genomics, Vineland Research and Innovation Centre, Vineland Station, ON, Canada L0R 2E0; ^3^Cereal Research Centre, Agriculture and Agri-Food Canada, Winnipeg, MB, Canada R3T 2M9

## Abstract

The decreasing cost along with rapid progress in next-generation sequencing and related bioinformatics computing resources has facilitated large-scale discovery of SNPs in various model and nonmodel plant species. Large numbers and genome-wide availability of SNPs make them the marker of choice in partially or completely sequenced genomes. Although excellent reviews have been published on next-generation sequencing, its associated bioinformatics challenges, and the applications of SNPs in genetic studies, a comprehensive review connecting these three intertwined research areas is needed. This paper touches upon various aspects of SNP discovery, highlighting key points in availability and selection of appropriate sequencing platforms, bioinformatics pipelines, SNP filtering criteria, and applications of SNPs in genetic analyses. The use of next-generation sequencing methodologies in many non-model crops leading to discovery and implementation of SNPs in various genetic studies is discussed. Development and improvement of bioinformatics software that are open source and freely available have accelerated the SNP discovery while reducing the associated cost. Key considerations for SNP filtering and associated pipelines are discussed in specific topics. A list of commonly used software and their sources is compiled for easy access and reference.

## 1. Introduction 

Molecular markers are widely used in plant genetic research and breeding. Single Nucleotide Polymorphisms (SNPs) are currently the marker of choice due to their large numbers in virtually all populations of individuals. The applications of SNP markers have clearly been demonstrated in human genomics where complete sequencing of the human genome led to the discovery of several million SNPs [1] and technologies to analyze large sets of SNPs (up to 1 million) have been developed. SNPs have been applied in areas as diverse as human forensics [2] and diagnostics [3], aquaculture [4], marker assisted-breeding of dairy cattle [5], crop improvement [6], conservation [7], and resource management in fisheries [8]. Functional genomic studies have capitalized upon SNPs located within regulatory genes, transcripts, and Expressed Sequence Tags (ESTs) [9, 10]. Until recently large scale SNP discovery in plants was limited to maize,* Arabidopsis*, and rice [11–15]. Genetic applications such as linkage mapping, population structure, association studies, map-based cloning, marker-assisted plant breeding, and functional genomics continue to be enabled by access to large collections of SNPs. *Arabidopsis thaliana* was the first plant genome sequenced [16] followed soon after by rice [17, 18]. In the year 2011 alone, the number of plant genomes sequenced doubled as compared to the number sequenced in the previous decade, resulting in currently, 31 and counting, publicly released sequenced plant genomes (http://www.phytozome.net/). With the ever increasing throughput of next-generation sequencing (NGS), *de novo* and reference-based SNP discovery and application are now feasible for numerous plant species.

Sequencing refers to the identification of the nucleotides in a polymer of nucleic acids, whether DNA or RNA. Since its inception in 1977, sequencing has brought about the field of genomics and increased our understanding of the organization and composition of plant genomes. Tremendous improvements in sequencing have led to the generation of large amounts of DNA information in a very short period of time [19]. The analyses of large volumes of data generated through various NGS platforms require powerful computers and complex algorithms and have led to a recent expansion of the bioinformatics field of research. This book chapter focuses on the *a priori* discovery of SNPs through NGS, bioinformatics tools and resources, and the various downstream applications of SNPs.

## 2. History and Evolution of Sequencing ****Technologies

### 2.1. Invention of Sequencing

In 1977, two sequencing methods were developed and published. The Sanger method is a sequencing-by-synthesis (SBS) method that relies on a combination of deoxy- and dideoxy-labeled chain terminator nucleotides [20]. The first complete genome sequencing, that of bacteriophage *phi X174*, was achieved that same year using this pioneering method [21]. The chemical modification followed by cleavage at specific sites method also published in 1977 [22] quickly became the less favored of the two methods because of its technical complexities, use of hazardous chemicals, and inherent difficulty in scale-up. In contrast, the Sanger method, for which Frederick Sanger was awarded his second Nobel Prize in chemistry in 1980, was quickly adopted by the biotechnology industry which implemented it using a broad array of chemistries and detection methods [19].

### 2.2. Sequencing Technologies

In the last decade, new sequencing technologies have outperformed Sanger-based sequencing in throughput and overall cost, if not quite in sequence length and error rate [23]. This section will focus on the three main NGS platforms as well as the two main third-generation sequencing (TGS) platforms, their throughput and relative cost. We made every effort to ensure the accuracy of the data at the time of submission. However, the cost and throughput of these sequencing platforms change rapidly and, as such, our analysis only represents a snapshot in time. The flux of innovation in this field imposes a need for constant assessment of the technologies' potentials and realignment of research goals. 

#### 2.2.1. Roche (454) Sequencing

Pyrosequencing was the first of the new highly parallel sequencing technologies to reach the market [24]. It is commonly referred to as 454 sequencing after the name of the company that first commercialized it. It is an SBS method where single fragments of DNA are hybridized to a capture bead array and the beads are emulsified with regents necessary to PCR amplifying the individually bound template. Each bead in the emulsion acts as an independent PCR where millions of copies of the original template are produced and bound to the capture beads which then serve as the templates for the subsequent sequencing reaction. The individual beads are deposited into a picotiter plate along with DNA polymerase, primers, and the enzymes necessary to create fluorescence through the consumption of inorganic phosphate produced during sequencing. The instrument washes the picotiter plate with each of the DNA bases in turn. As template-specific incorporation of a base by DNA polymerase occurs, a pyrophosphate (PPi) is produced. This pyrophosphate is detected by an enzymatic luminometric inorganic pyrophosphate detection assay (ELIDA) through the generation of a light signal following the conversion of PPi into ATP [25]. Thus, the wells in which the current nucleotides are being incorporated by the sequencing reaction occurring on the bead emit a light signal proportional to the number of nucleotides incorporated, whereas wells in which the nucleotides are not being incorporated do not. The instrument repeats the sequential nucleotide wash cycle hundreds of times to lengthen the sequences. The 454 GS FLX Titanium XL^+^ platform currently generates up to 700 MB of raw 750 bp reads in a 23 hour run. The technology has difficulty quantifying homopolymers resulting in insertions/deletions and has an overall error rate of approximately 1%. Reagent costs are approximately $6,200 per run [26].

#### 2.2.2. Illumina Sequencing

Illumina technology, acquired by Illumina from Solexa, followed the release of 454 sequencing. With this sequencing approach, fragments of DNA are hybridized to a solid substrate called a flow cell. In a process called bridge amplification, the bound DNA template fragments are amplified in an isothermal reaction where copies of the template are created in close proximity to the original. This results in clusters of DNA fragments on the flow cell creating a “lawn” of bound single strand DNA molecules. The molecules are sequenced by flooding the flow cell with a new class of cleavable fluorescent nucleotides and the reagents necessary for DNA polymerization [27]. A complementary strand of each template is synthesized one base at a time using fluorescently labeled nucleotides. The fluorescent molecule is excited by a laser and emits light, the colour of which is different for each of the four bases. The fluorescent label is then cleaved off and a new round of polymerization occurs. Unlike 454 sequencing, all four bases are present for the polymerization step and only a single molecule is incorporated per cycle. The flagship HiSeq2500 sequencing instrument from Illumina can generate up to 600 GB per run with a read length of 100 nt and 0.1% error rate. The Illumina technique can generate sequence from opposite ends of a DNA fragment, so called paired-end (PE) reads. Reagent costs are approximately $23,500 per run [26].

#### 2.2.3. Applied Biosystems (SOLiD) Sequencing

The SOLiD system was jointly developed by the Harvard Medical School and the Howard Hughes Medical Institute [28]. The library preparation in SOLiD is very similar to Roche/454 in which clonal bead populations are prepared in microreactors containing DNA template, beads, primers, and PCR components. Beads that contain PCR products amplified by emulsion PCR are enriched by a proprietary process. The DNA templates on the beads are modified at their 3′ end to allow attachment to glass slides. A primer is annealed to an adapter on the DNA template and a mixture of fluorescently tagged oligonucleotides is pumped into the flow cell. When the oligonucleotide matches the template sequence, it is ligated onto the primer and the unincorporated nucleotides are washed away. A charged couple device (CCD) camera captures the different colours attached to the primer. Each fluorescence wavelength corresponds to a particular dinucleotide combination. After image capture, the fluorescent tag is removed and new set of oligonucleotides are injected into the flow cell to begin the next round of DNA ligation [19]. This sequencing-by-ligation method in SOLiD-5500x1 platform generates up to 1,410 million PE reads of 75 + 35 nt each with an error rate of 0.01% and reagent cost of approximately $10,500 per run [26].

Although widely accepted and used, the NGS platforms suffer from amplification biases introduced by PCR and dephasing due to varying extension of templates. The TGS technologies use single molecule sequencing which eliminates the need for prior amplification of DNA thus overcoming the limitations imposed by NGS. The advantages offered by TGS technology are (i) lower cost, (ii) high throughput, (iii) faster turnaround, and (iv) longer reads [19, 29]. The TGS can broadly be classified into three different categories: (i) SBS where individual nucleotides are observed as they incorporate (Pacific Biosciences single molecule real time (SMART), Heliscope true single molecule sequencing (tSMS), and Life Technologies/Starlight and Ion Torrent), (ii) nanopore sequencing where single nucleotides are detected as they pass through a nanopore (Oxford/Nanopore), and (iii) direct imaging of individual molecules (IBM). 

#### 2.2.4. Helicos Biosciences Corporation (Heliscope) Sequencing

Heliscope sequencing involves DNA library preparation and DNA shearing followed by addition of a poly-A tail to the sheared DNA fragments. These poly-A tailed DNA fragments are attached to flow cells through poly-T anchors. The sequencing proceeds by DNA extension with one out of 4 fluorescent tagged nucleotides incorporated followed by detection by the Heliscope sequencer. The fluorescent tag on the incorporated nucleotide is then chemically cleaved to allow subsequent elongation of DNA [30]. Heliscope sequencers can generate up to 28 GB of sequence data per run (50 channels) with maximum read length of 55 bp at ~99% accuracy [31]. The cost per run per channel is approximately $360.

#### 2.2.5. Pacific Biosciences SMART Sequencing

The Pacific Biosciences sequencer uses glass anchored DNA polymerases which are housed at the bottom of a zero-mode waveguide (ZMW). DNA fragments are added into the ZMW chamber with the anchored DNA polymerase and nucleotides, each labeled with a different colour fluorophore, and are diffused from above the ZMW. As the nucleotides circulate through the ZMW, only the incorporated nucleotides remain at the bottom of the ZMW while unincorporated nucleotides diffuse back above the ZMW. A laser placed below the ZMW excites only the fluorophores of the incorporated nucleotides as the ZMW entraps the light and does not allow it to reach the unincorporated nucleotides above [32]. The Pacific Biosciences sequencers can generate up to 140 MB of sequences per run (per smart cell) with reads of 2.5 Kbp at ~85% accuracy. The cost per run per smart cell is approximately $600.

Among the TGS technologies, Pacific Biosciences SMART and Heliscope tSMS have been used in characterizing bacterial genomes and in human-disease-related studies [31]; however, TGS has yet to be capitalized upon in plant genomes. The Heliscope generates short reads (55 bp) which may cause ambiguous read mapping due to the presence of paralogous sequences and repetitive elements in plant genomes. The Pacific Biosciences reads have high error rates which limit their direct use in SNP discovery. However, their long reads offer a definite advantage to fill gaps in genomic sequences and, at least in bacterial genomes, NGS reads have proven capable of “correcting” the base call errors of this TGS technology [33–36]. Hybrid assemblies incorporating short (Illumina, SOLiD), medium (454/Roche), and long reads (Pac-Bio) have the potential to yield better quality reference genomes and, as such, would provide an improved tool for SNP discovery.

The choice of a sequencing strategy must take into account the research goals, ability to store and analyze data, the ongoing changes in performance parameters, and the cost of NGS/TGS platforms. Some key considerations include cost per raw base, cost per consensus base, raw and consensus accuracy of bases, read length, cost per read, and availability of PE or single end reads. The pre- and postprocessing protocols such as library construction [37] and pipeline development and implementation for data analysis [38] are also important.

### 2.3. RNA and ChIP Sequencing

Genome-wide analyses of RNA sequences and their qualitative and quantitative measurements provide insights into the complex nature of regulatory networks. RNA sequencing has been performed on a number of plant species including *Arabidopsis* [39], soybean [40], rice [41], and maize [42] for transcript profiling and detection of splice variants. RNA sequencing has been used in *de novo* assemblies followed by SNP discovery performed in nonmodel plants such as *Eucalyptus grandis* [43], *Brassica napus* [44], and* Medicago sativa* [45]. 

RNA deep-sequencing technologies such as digital gene expression [46] and Illumina RNASeq [47] are both qualitative and quantitative in nature and permit the identification of rare transcripts and splice variants [48]. RNA sequencing may be performed following its conversion into cDNA that can then be sequenced as such. This method is, however, prone to error due to (i) the inefficient nature of reverse transcriptases (RTs) [49], (ii) DNA-dependent DNA polymerase activity of RT causing spurious second strand DNA [50], and (iii) artifactual cDNA synthesis due to template switching [51]. Direct RNA sequencing (DRS) developed by Helicos Biosciences Corporation is a high throughput and cost-effective method which eliminates the need for cDNA synthesis and ligation/amplification leading to improved accuracy [52].

Chromatin immunoprecipitation (ChIP) is a specialized sequencing method that was specifically designed to identify DNA sequences involved in *in vivo* protein DNA interaction [53]. ChIP-sequencing (ChIP-Seq) is used to map the binding sites of transcription factors and other DNA binding sites for proteins such as histones. As such, ChIP-Seq does not aid SNP discovery, but the availability of SNP data along with ChIP-Seq allows the study of allele-specific states of chromatin organization. Deep sequence coverage leading to dense SNP maps permits the identification of transcription factor binding sites and histone-mediated epigenetic modifications [54]. ChIP-Seq can be performed on serial analysis of gene expression (SAGE) tags or PE using Sanger, 454, and Illumina platforms [55, 56]. 

The DNA, RNA, and ChIP-Seq data is analysed using a reference sequence if available or, in the absence of such reference, it requires *de novo* assembly, all of which is performed using specialized software, algorithms, pipelines, and hardware. 

## 3. Computing Resources for Sequence Assembly

The next-generation platforms generate a considerable amount of data and the impact of this with respect to data storage and processing time can be overlooked when designing an experiment. Bioinformatics research is constantly developing new software and algorithms, data storage approaches, and even new computer architectures to better meet the computation requirements for projects incorporating NGS. This chapter describes the state-of-the-art with respect to software for NGS alignment and analysis at the time of writing.

### 3.1. Software for Sequence Analysis

Both commercial and noncommercial sequence analysis software are available for Windows, Macintosh, and Linux operating systems. NGS companies offer proprietary software such as consensus assessment of sequence and variation (Cassava) for Illumina data and Newbler for 454 data. Such software tend to be optimized for their respective platform but have limited cross applicability to the others. Web-based portals such as Galaxy [57] are tailored to a multitude of analyses, but the requirement to transfer multigigabyte sequence files across the internet can limit its usability to smaller datasets. Commercially available software such as CLC-Bio (http://www.clcbio.com/) and SeqMan NGen (http://www.dnastar.com/t-sub-products-genomics-seqman-ngen.aspx) provide a friendly user interface, are compatible with different operating systems, require minimal computing knowledge, and are capable of performing multiple downstream analyses. However, they tend to be relatively expensive, have narrow customizability, and require locally available high computing power. A recent review by Wang et al. [58] recommends Linux-based programs because they are often free, not specific to any sequencing platform, and less computing power hungry and, as a consequence, tend to perform faster. Flexibility in the parameter's choice for read assembly is another major advantage. However, most biologists are unfamiliar with Linux operating systems, its structure and command lines, thereby imposing a steep learning curve for adoption. Linux-based software such as Bowtie [59], BWA [60], and SOAP2/3 [61] have been used widely for the analysis of NGS data. Other software may not have gained broad acceptance but may have unique features worth noting. For reviews on NGS software, see Li and Homer [62], Wang et al. [58], and Treangen and Salzberg [63]. Characteristics of the most common NGS software and their attributes are listed in Table 1, and their download information can be found in Table 4. 

### 3.2. Consideration for Software Selection

In selecting software for NGS data analysis one must consider, among other things, the sequencing platform, the availability of a reference genome, the computing and storage resources necessary, and the bioinformatics expertise available. Algorithms used for sequence analysis have matured significantly but may still require computing power beyond what is currently available in most genomics facilities and/or long processing time. For example, in aligning 2 × 13,326,195 paired-end reads (76 bp) from The Cancer Genome Atlas project (SRR018643) [64], SHRiMP [65] took 1,065 hrs with a peak memory footprint of 12 gigabytes to achieve the mapping of 81% of the reads to the human genome reference whereas Bowtie used 2.9 gigabytes of memory, a run time of 2.2 hrs but only achieved a 67% mapping rate [58]. Both time and memory become critical when dealing with a very large NGS dataset. Fast and memory efficient sequence mapping seems to be preferred over slower, memory demanding software even at the cost of a reduced mapping rate. It should be noted that a higher percentage of mapped reads is not a strict measure of quality because it may be indicative of a higher level of misaligned reads or reads aligned against repetitive elements, features that are not desirable [63]. 

In the absence of a reference genome, *de novo* assembly of a plant genome is achieved using sequence information obtained through a combination of Sanger and/or NGS of bacterial artificial chromosome (BAC) clones, or by whole genome shotgun (WGS) with NGS [66]. *De novo* assemblies are time consuming and require much greater computing power than read mapping onto a reference genome. The assembly accuracy depends in part on the read length and depth as well as the nature of the sequenced genome. The genomes of *Arabidopsis thaliana* [16], rice [67], and maize [68] were generated using a BAC-by-BAC approach while poplar [69], grape [70], and sorghum [71] genomic sequences were obtained through WGS. All genomes sequenced to date are fragmented to varying degrees because of the inability of sequencing technologies and bioinformatics algorithms to assemble through highly conserved repetitive elements. A list of current plant genome sequencing projects, their sequencing strategies, and status from standard draft to finished can be found in the review by Feuillet et al. [72].

Software programs such as Mira [73], SOAPdenovo [74], ABySS [75], and Velvet [76] have been used for *de novo *assembly. MIRA is well documented and can be readily customized, but it requires substantial computing memory and is not suited for large complex genomes. Of the freely available software, SOAPdenovo is one of the fastest read assembly programs and it uses a comparatively moderate amount of computing memory. The assembly generated by SOAPdenovo can be used for SNP discovery using SOAPsnp as implemented for the apple genome [77]. ABySS can be deployed on a computer cluster. It requires the least amount of memory and can be used for large genomes. Velvet requires the largest amount of memory. It can use mate-pair information to resolve and correct assembly errors. 

## 4. SNP Discovery

The most common application of NGS is SNP discovery, whose downstream usefulness in linkage map construction, genetic diversity analyses, association mapping, and marker-assisted selection has been demonstrated in several species [78]. NGS-derived SNPs have been reported in humans [79], *Drosophila* [80], wheat [81, 82], eggplant [83], rice [84–86], *Arabidopsis* [87, 88], barley [14, 89], sorghum [90], cotton [91], common beans [78], soybean [92], potato [93], flax [94], *Aegilops tauschii* [95], alfalfa [96], oat [97], and maize [98] to name a few. 

SNP discovery using NGS is readily accomplished in small plant genomes for which good reference genomes are available such as rice and *Arabidopsis* [86, 99]. Although SNP discovery in complex genomes without a reference genome such as wheat [81, 82], barley [14, 89], oat [97], and beans [78] can be achieved through NGS, several challenges remain in other nonmodel but economically important crops. The presence of repeat elements, paralogs, and incomplete or inaccurate reference genome sequences can create ambiguities in SNP calling [63]. NGS read mapping can also suffer from sequencing error (erroneous base calling) and misaligned reads. The following section focuses on programs tailored for SNP discovery and emphasizes some of the precautions and considerations to minimize erroneous SNP calling.

### 4.1. Software and Pipelines for SNP Discovery

**Figure 1:**
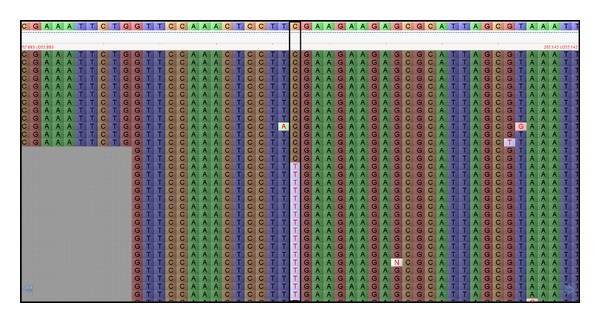
Graphical user interface of Tablet, an assembly visualization program, displays the reference genome on top and the mapped reads with color-coded SNPs on the bottom.

In theory, a SNP is identified when a nucleotide from an accession read differs from the reference genome at the same nucleotide position. In the absence of a reference genome, this is achieved by comparing reads from different genotypes using *de novo *assembly strategies [95]. Read assembly files generated by mapping programs are used to perform SNP calling. In practice, various empirical and statistical criteria are used to call SNPs, such as a minimum and maximum number of reads considering the read depth, the quality score and the consensus base ratio for examples [95]. Thresholds for these criteria are adjusted based on the read length and the genome coverage achieved by the NGS data. In assemblies generated allowing single nucleotide variants and insertions/deletions (indels), a list of SNP and indel coordinates is generated and the read mapping results can be visualized using graphical user interface programs such as Tablet [100] (Figure 1), SNP-VISTA [101], or Savant [102] (refer to Table 4 for download information). Tablet has a user-friendly interface and is widely used because it supports a wide array of commonly used file formats such as SAM, BAM, SOAP, ACE, FASTQ, and FASTA generated by different read assemblers such as Bowtie, BWA, SOAP, MAQ, and SeqMan NGen. It displays contig overview, coverage information, read names and it allows searching for specific coordinates on scaffolds. 

Broadly used SNP calling software include Samtools [103], SNVer [104], and SOAPsnp [74]. Samtools is popular because of its various modules for file conversion (SAM to BAM and vice-versa), mapping statistics, variant calling, and assembly visualization. Recently, SOAPsnp has gained popularity because of its tight integration with SOAP aligner and other SOAP modules which are constantly upgraded and provide a one stop shop for the sequencing analysis continuum. Variant calling algorithms such as Samtools and SNVer can be used as stand-alone programs or incorporated into pipelines for SNP calling. Reviews of SNP calling software have been published [63, 105]. Some of the main features of the current commonly used software are listed in Table 2 (refer to Table 4 for download information). 

### 4.2. SNP Discovery from Multiple Individuals and Complex Genomes

SNP discovery is more robust when multiple and divergent genotypes are used simultaneously, creating the necessary basis to capture the genetic variability of a species. Large parts of plant genomes consist of repetitive elements [106] which can cause spurious SNP calling by erroneous read mapping to paralogous repeat element sequences. In polyploid genomes such as cotton (allotetraploid), homoeologous sequences can cause similar misalignment [91]. Improved read assembly and filtering of SNPs become even more important factors for accurate SNP calling in these cases because they can mitigate the effects of errors caused by paralogs and homoeologs. 

Read assembly algorithms such as Bowtie and SOAP as well as variant calling/genotyping softwares such as GATK [107] are rapidly evolving to accommodate an ever increasing number of reads, increased read length, nucleotide quality values, and mate-pair information of PE reads. Assembly programs such as Novoalign (http://www.novocraft.com/main/index.php) and STAMPY [108], although memory and time intensive, are highly sensitive for simultaneous mapping of short reads from multiple individuals [105]. 

SNP calls can be significantly improved using filtering criteria that are specific to the genome characteristics and the dataset. For instance, projects aimed at resequencing can compare different datasets from the same genotype and thus eliminate data with large discrepancies. This strategy identifies the most common sources of error and is applied in the 1000 genome project [109]. Reduced representation libraries (RRLs), that is, sequencing an enriched subset of a genome by eliminating a proportion of its repetitive fractions [79], reduce the probability of misalignments to repeats and thus potential downstream erroneous SNP calling. Filtering criteria that can improve SNP accuracy include (i) a minimum read depth (often ≥3 per genotype), (ii) >90% nucleotides within a genotype having identical call at a given position (~<10% sequencing error), (iii) a read depth ≤ mean of the sequence depth over the entire mapping assembly, (iv) the elimination of ribosomal DNA and other repetitive elements in the 50 nt flanking any SNP call, and (v) masking of homopolymer SNPs with a given base string length (often ≥2). Additionally, in polyploid species, separate assembly of homoeologs using stringent mapping parameters is often essential for genome-wide SNP identification to avoid spurious SNP calls caused by erroneous homoeologous read mapping [91].

### 4.3. SNP Validation

**Figure 2:**
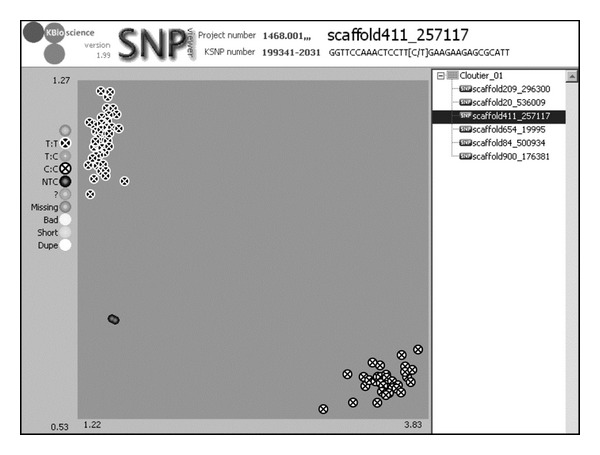
Validation of a T/C SNP by a KASPar assay (KBiosciences, Herts, England). Genotypes with a “T” are represented by black dots with a white cross clustered in the upper left and those with a “C” by white dots with a black cross in the bottom right cluster. The two black dots near the bottom left are negative controls. No heterozygous individuals were present in this population.

Prior to any SNP applications, the discovered SNPs must be validated to identify the true SNPs and get an idea of the percentage of potentially false SNPs resulting from an SNP discovery exercise. The need for validation arises because a proportion of the discovered SNPs could have been wrongly called for various reasons including those outlined above. SNP validation can be accomplished using a variety of material such as a biparental segregating population or a diverse panel of genotypes. Usually a small subset of the SNPs is used for validation through assays such as the Illumina Goldengate [110], KBiosciences Competitive Allele*­*Specific-PCR SNP genotyping system (KASPar) (http://www.lgcgenomics.com/) or the High Resolution Melting (HRM) curve analysis. Validation can serve as an iterative and informative process to modify and optimize the SNP filtering criteria to improve SNP calling. For example, a subset of 144 SNPs from a total of 2,113,120 SNPs were validated using the Goldengate assay on 160 accessions in apple [77]. Another example is illustrated in Figure 2 where a KASPar assay was performed on 92 genotypes from a segregating population illustrating the validation of a single “T/C” SNP in two distinct clusters. Other validation strategies used in nonmodel organisms are tabulated in Garvin et al. [111]. With the continuously competitive pricing of NGS, genotyping-by-sequencing (GBS) is becoming a viable SNP validation method. Either biparental segregating populations or a collection of diverse genotypes can be sequenced at a reasonable cost using indexing, that is, combining multiple independently tagged genotypes in a single NGS run to obtain genome-wide or reduced representation genome sequences at a lower coverage but potentially validating a much larger number of SNPs than the methods described above. Sequencing of segregating populations or diverse genotypes may also lead to the discovery of additional SNPs. 

The two major factors affecting the SNP validation rate are sequencing and read mapping errors as discussed above. NGS platforms have different levels of sequencing accuracies, and this may be the most important factor determining the variation in the validation, from 88.2% for SOLiD followed by Illumina at 85.4% and Roche 454 at 71% [95]. The SNP validation rates can be improved using RRL for SNP discovery and choosing SNPs within the nonrepetitive sequences including predicted single copy genes and single copy repeat junctions shown to have high validation rates [95]. 

## 5. SNP Genotyping 

SNP genotyping is the downstream application of SNP discovery to identify genetic variations. SNP applications include phylogenic analysis, marker-assisted selection, genetic mapping of quantitative trait loci (QTL), bulked segregant analysis, genome selection, and genome-wide association studies (GWAS). The number of SNPs and individuals to screen are of primary importance in choosing an SNP genotyping assay, though cost of the assay and/or equipment and the level of accuracy are also important considerations. 

Illumina Goldengate is a commonly used genotyping assay because of its flexibility in interrogating 96 to 3,072 SNP loci simultaneously (http://www.illumina.com/). HRM analysis is suitable for a few to an intermediate number of SNPs and can be performed within a typical laboratory setting. KASPar and SNPline genotyping systems (http://www.lgcgenomics.com/) can be used for genotyping a few to thousands of SNPs in a laboratory setting. The SNPline system is available in SNPlite or SNPline XL versions to allow flexibility in sample number and SNP assays. The iPLEX Gold technology developed by Sequenom (http://www.sequenom.com/) is based on the MassARRAY system which uses primer extension chemistry and matrix-assisted laser desorption/ionisation-time of flight (MALDI-TOF) mass spectrometry for genotyping. 

The iPLEX Gold system has gained acceptance due to its high precision and cost-effective implementation. High throughput chip-based genotyping assays such as the Affymetrix GeneChip arrays (http://www.affymetrix.com/estore/) and the Illumina BeadChips (http://www.illumina.com/) are capable of validating up to a million SNPs per reaction across an entire genome. Detailed analyses of SNP genotyping assays and their features are reviewed in Tsuchihashi and Dracopoli [112], Sobrino and Carracedo [113], Giancola et al. [114], Kim and Misra [115], Gupta et al. [116], and Ragoussis [117]. A list of the most commonly used genotyping assays describing the assay type, technology, throughput, multiplexing ability, and relative scalability can be found in Table 3. 

Array-based technologies such as Infinium and Goldengate substantially improved SNP genotyping efficiency, but they are species-specific, expensive to design and require specific equipment and chemistry. PCR and primer extension technologies like KASPar and Taqman (http://www.lifetechnologies.com/global/en/home.html) are limited by their low SNP throughput but can be useful to assay a large number of genotypes with few SNPs. NGS technologies have become viable for genotyping studies and may offer advantages over other genotyping methods in cost and efficiency. 

### 5.1. Genotyping-by-Sequencing (GBS)

There have been a number of approaches developed that use complexity reduction strategies to lower the cost and simplify the discovery of SNP markers using NGS, RNA-Seq, complexity reduction of polymorphic sequences (CRoPS), restriction-site-associated DNA sequencing (RAD-Seq), and GBS [118]. Of these methodologies GBS holds the greatest promise to serve the widest base of plant researchers because of its ability to allow simultaneous marker discovery and genotyping with low cost and a simple molecular biology workflow. Briefly, GBS involves digesting the genome of each individual in a population to be studied with a restriction enzyme [119]. One unique and one common adapter are ligated to the fragments and a PCR is carried out which is biased towards amplifying smaller DNA fragments. The resulting PCR products are then pooled and sequenced using an Illumina platform. The amplicons are not fragmented so only the ends of the PCR products are sequenced. The unique adapter acts as an ID tag so sequencing reads can be associated with an individual. The technique can be applied to species with or without a reference genome. The choice of enzyme has an effect on the number of markers identified and the amount of sequence coverage required. The more frequent the restriction recognition site, the higher the number of fragments and therefore more potential markers. Use of more frequent cutters may necessitate greater amounts of sequencing depending on the application. Poland et al. [120] recently demonstrated the use of two restriction enzymes to perform GBS in bread wheat, a hexaploid genome. 

GBS has the potential to be a truly revolutionary technology in the arena of plant genomics. It brings high density genotyping to the vast majority of plant species that, until now, have had almost no investment in genomics resources. With little capital investment requirement and an affordable per sample cost, all plant researchers now have powerful genomic and genetic methodologies available to them. Uses of GBS include applications in marker discovery, phylogenetics, bulked segregant analysis, QTL mapping in biparental lines, GWAS, and genome selection. GBS can also be applied to fine mapping in candidate gene discovery and be used to generate high-density SNP genetic maps to assist in *de novo* genome assembly. We predict tremendous advances in functional genomics and plant breeding from the implementation of GBS because it is truly a democratizing application for NGS in nonmodel plant systems.

## 6. Applications of SNPS

NGS and SNP genotyping technologies have made SNPs the most widely used marker for genetic studies in plant species such as *Arabidopsis* [121] and rice [122]. SNPs can help to decipher breeding pedigree, to identify genomic divergence of species to elucidate speciation and evolution, and to associate genomic variations to phenotypic traits [85]. The ease of SNP development, reasonable genotyping costs, and the sheer number of SNPs present within a collection of individuals allow an assortment of applications that can have a tremendous impact on basic and applied research in plant species.

### 6.1. SNPs in Genetic Mapping

A genetic map refers to the arrangement of traits, genes, and markers relative to each other as measured by their recombination frequency. Genetic maps are essential tools in molecular breeding for plant genetic improvement as they enable gene localization, map-based cloning, and the identification of QTL [123]. SNPs have greatly facilitated the production of much higher density maps than previous marker systems. SNPs discovered using RNA-Seq and expressed sequence tags (ESTs) have the added advantage of being gene specific [124]. Their high abundance and rapidly improving genotyping technologies make SNPs an ideal marker type for generating new genetic maps as well as saturating existing maps created with other markers. Most SNPs are biallelic thereby having a lower polymorphism information content (PIC) value as compared to most other marker types which are often multiallelic [125]. The limited information associated with their biallelic nature is greatly compensated by their high frequency, and a map of 700–900 SNPs has been found to be equivalent to a map of 300–400 simple sequence repeat (SSR) markers [125]. SNP-based linkage maps have been constructed in many economically important species such as rice [126], cotton [91] and *Brassica* [127]. The identification of candidate genes for flowering time in *Brassica* [127] and maize [128] are practical examples of gene discovery through SNP-based genetic maps. 

### 6.2. Genome-Wide Association Mapping

Association mapping (AM) panels provide a better resolution, consider numerous alleles, and may provide faster marker-trait association than biparental populations [129]. AM, often referred to as linkage disequilibrium (LD) mapping, relies on the nonrandom association between markers and traits [130]. LD can vary greatly across a genome. In low LD regions, high marker saturation is required to detect marker-trait association, hence the need for densely saturated maps. In general, GWASs require 10,000–100,000 markers applied to a collection of genotypes representing a broad genetic basis [130]. 

In the past few years, NGS technologies have led to the discovery of thousands, even millions of SNPs, and novel application platforms have made it possible to produce genome-wide haplotypes of large numbers of genotypes, making SNPs the ideal marker for GWASs. So far, 951 GWASs have been reported in humans (http://www.bing.com/search?q=www.genome.gov%2Fgwastudies%2F&src=ie9tr). In plants, such a study was first reported in *Arabidopsis* for flowering time and pathogen-resistance genes [131]. A GWAS performed in rice using ~3.6 million SNPs identified genomic regions associated with 14 agronomic traits [132]. The genetic structure of northern leaf blight, southern leaf blight, and leaf architecture was studied using ~1.6 million SNPs in maize [133–135]. SNP-based GWAS was also performed on species such as barley for which a reference genome sequence is not available [136]. Soto-Cerda and Cloutier [137] have reviewed the concepts, benefits, and limitations of AM in plants.

### 6.3. Evolutionary Studies

SSRs and mitochondrial DNA have been used in evolutionary studies since the early 1990s [138]. However, the biological inferences from results of these two marker types may be misinterpreted due to homoplasy, a phenomenon in which similarity in traits or markers occurs due to reasons other than ancestry, such as convergent evolution, evolutionary reversal, gene duplication, and horizontal gene transfer [139]. The advantage of SNPs over microsatellites and mitochondrial DNA resides in the fact that SNPs represent single base nucleotide substitutions and, as such, they are less affected by homoplasy because their origin can be explained by mutation models [140]. SNPs have been employed to quantify genetic variation, for individual identification, to determine parentage relatedness and population structure [138]. Seed shattering (or loss thereof) has been associated with an SNP through a GWAS aimed at unraveling the evolution of rice that led to its domestication [141]. SNPs have also been used to study the evolution of genes such as *WAG-2* in wheat [142]. Algorithms such as neighbor-joining and maximum likelihood implemented in the PHYLIP [143] and MEGA [144] software are commonly used to generate phylogenetic trees. 

The main advantage of SNPs is unquestionably their large numbers. As with all marker systems the researcher must be aware of ascertainment biases that exist in the panel of SNPs being used. These biases exist because SNPs are often developed from examining a small group of individuals and selecting the markers that maximize the amount of polymorphism that can be detected in the population used. This results in a collection of markers that sample only a fraction of the diversity that exists in the species but that are nevertheless used to infer relatedness and determine genetic distance for whole populations. Ideally, a set of SNP markers randomly distributed throughout the genome would be developed for each population studied. GBS moves us closer to this goal by incorporating simultaneous discovery of SNPs and genotyping of individuals. With this approach genome sample bias remains but can be mitigated by careful restriction enzyme selection.

## 7. Future Perspectives

SNP discovery incontestably made a quantum leap forward with the advent of NGS technologies and large numbers of SNPs are now available from several genomes including large and complex ones (see Section 4). Unlike model systems such as humans and *Arabidopsis*, SNPs from crop plants remain limited for the time being, but broad access to reasonable cost NGS promises to rapidly increase the production of reference genome sequences as well as SNP discovery. Many issues remain to be addressed, such as the ascertainment bias of popular biparental populations and the low validation rate of some array-based genotyping platforms [145]. The area of epigenetic regulation of various genome components can be better understood as accurate and deeper sequencing is achieved. RNA and ChIP-sequencing projects, similar to RNA-Seq in the nonmodel plant sweet cherry to identify SNPs and haplotypes [146], can be undertaken to study functional genomics. A great deal of knowledge that is still elusive about the noncoding and repetitive elements can be determined with the next wave of modern and efficient sequencing technologies.

The first (Sanger) and the second (next) generation sequencing technologies have enabled researchers to characterize DNA sequence variation, sequence entire genomes, quantify transcript abundance, and understand mechanisms such as alternative splicing and epigenetic regulation [29]. 

Numerous plant genomes are now sequenced at various levels of completion and many more are underway [72]. The NGS technologies have made SNP discovery affordable even in complex genomes and the technologies themselves have improved tremendously in the past decade. Improvements in TGS promise synergies with NGS technologies to further assist our understanding of plant genetics and genomics. NGS has revolutionized genomics-related research, and it is our belief that the NGS-enabled discoveries will continue in the next decade. 

## Figures and Tables

**Table 1:** List of most cited/used software for sequence assembly of NGS data. Source locations for these software are compiled in Table 4.

Name (current version)	Assembly type	Supported parameters				Output format	Platform
	(algorithm)	Color space	Read length	Gapped alignment	Paired-end		
CLC-Bio^1^	Reference^2^	Yes	Arbitrary	Yes	Yes	CLC-Bio	Linux/Windows/Mac OS X
SeqMan NGen^1^	Reference^2^	Yes	Arbitrary	Yes	Yes	ACE, BAM	Windows/Mac OS X
NextGENe^1^	Reference^2^	Yes	Arbitrary	Yes	Yes	Next GENe	Windows/Mac OS X
Bowtie (2)	Reference (FM-index)	Yes	Arbitrary	Yes	Yes	SAM	Linux/Windows/Mac OS X
BWA	Reference (FM-index)	Yes	Arbitrary	Yes	Yes	SAM	Linux
SOAP (3)	Reference (FM-index)	Yes	Arbitrary	No	Yes	SOAP2/3	Linux
MAQ (0.6.6)	Reference (Hashing reads)	Yes	≤127	Yes	Yes	MAQ	Linux/Solaris/Mac OS X
Novoalign (2.07.07)	Reference (Hashing reference)	Yes	Arbitrary	Yes	Yes	SAM	Linux/Mac OS X
Mosaik (1.1.0018)	Reference (Hashing reference)	Yes	Arbitrary	Yes	Yes	SAM	Linux/Windows/Mac OS X/Solaris
SHRiMP (2.2.2)	Reference (Hashing reference)	Yes	Arbitrary	Yes	Yes	SAM	Linux/Mac OS X
Mira (3.4)	Reference^2^	Yes	Arbitrary	Yes	Yes	FASTA, ACE	Linux

^1^Commercial software. ^2^Option for *de* *novo* assembly and modules included for variant calling.

**Table 2:** Commonly used NGS variant calling software. Download information for these software is compiled in Table 4. A more comprehensive list of variant calling programs is available at http://seqanswers.com/wiki/Software/list.

Software	Multisample support	Reference	Features	Platform
Samtools	Yes	Aligned reads	Include computation of genotype likelihoods and variant calling	Linux
SOAPsnp	No	Variant database	Part of SOAP3 for variant calling	Linux
GATK	Yes	Aligned reads	Include variant caller, SNP filter, and SNP quality calibrator	Linux
SNVer	Yes	Aligned reads	Fast variant caller, assigning SNP significance based on read depth	Windows, Linux, Mac OS X
SHORE	Yes	Aligned reads	Variant calling based on reference sequence even from other species	Linux, Mac OS X
MaCH	Yes	Genotype likelihoods	Variant calling with or without LD information	Windows, Linux, Mac OSX
IMPUTE2	Yes	Candidate SNPs and genotype likelihoods	Variant calling and linkage map-based SNP imputation	Windows, Linux, Mac OS X

**Table 3:** Commonly used genotyping platforms.

Name	Assay type	Technology	Throughput (samples)	Multiplexing	Relative scale (no. of SNP/no. of individuals)
Genechip	Hybridization	Oligo nucleotide array	96/5 days	Up to 18 × 10^6^	Small/large
Infinium II	Hybridization	Bead array	Up to 128/5 days	Up to 13 × 10^6^	Large/small-large
Goldengate	Primer extension-ligation	Bead array	172/3 days	Up to 3,072	Medium/large
iPlex	Primer extension	Mass spectrometry (MALDI-TOF)	3840/2.5 days	Up to 40	Medium/large
Taqman	PCR	Taqman probe	Up to 1536/day	Up to 256	Medium/medium
SNPlex	PCR	Capillary electrophoresis	Up to 1536/3 days	Up to 48	Medium/large
KASPar	PCR	FRET quenching oligos	Up to 96/day	—	Medium/large
Invader	Primer annealing/endonuclease digestion	FRET quenching oligos	Up to 384/day	Up to 200,000	Medium/large
HRM	PCR	Melting curve analysis	Up to 1536/day	—	Medium/large

**Table 4:** Download information of software used for NGS data.

Software	Source
Bowtie	http://bowtie-bio.sourceforge.net/bowtie2/index.shtml
BWA	http://bio-bwa.sourceforge.net/
SOAP	http://soap.genomics.org.cn/soap3.html#down2
MAQ	http://sourceforge.net/projects/maq/
Novoalign	http://www.novocraft.com/main/index.php
CLC-Bio Genomics	http://www.clcbio.com/index.php?id=1240
SeqManNGen	http://www.dnastar.com/t-products-seqman-ngen.aspx
NextGENe	http://softgenetics.com/NextGENe.html
Mosaik	http://bioinformatics.bc.edu/marthlab/Mosaik
SHRiMP	http://compbio.cs.toronto.edu/shrimp/
Mira	http://sourceforge.net/projects/mira-assembler/files/MIRA/stable/
Cassava	http://www.illumina.com/software/genome_analyzer_software.ilmn
Newbler	http://www.454.com/products/analysis-software/index.asp
Novoalign	http://www.novocraft.com/main/downloadpage.php
Tablet	http://bioinf.scri.ac.uk/tablet/
SNP-VISTA	http://genome.lbl.gov/vista/snpvista/
Samtools	http://sourceforge.net/projects/samtools/
Savant	http://genomesavant.com/savant/download.php
SOAPsnp	http://soap.genomics.org.cn/soapsnp.html
GATK	http://www.broadinstitute.org/gsa/wiki/index.php/
	The_Genome_Analysis_Toolkit
SNver	http://snver.sourceforge.net/
MaCH	http://www.sph.umich.edu/csg/abecasis/MACH/
IMPUTE2	http://mathgen.stats.ox.ac.uk/impute/impute_v2.html#
	download_impute2
MEGA	http://www.megasoftware.net/
PHYLIP	http://evolution.genetics.washington.edu/phylip.html
